# The Potential of Phytochemicals in Oral Cancer Prevention and Therapy: A Review of the Evidence

**DOI:** 10.3390/biom10081150

**Published:** 2020-08-06

**Authors:** Tzu-Ying Lee, Yu-Hsin Tseng

**Affiliations:** Department of Pediatrics, Kaohsiung Medical University Hospital, Kaohsiung Medical University, Kaohsiung 807, Taiwan; myleety@hotmail.com

**Keywords:** oral squamous cell carcinoma, oral cancer, phytochemicals

## Abstract

The etiological factors of oral cancer are complex including drinking alcohol, smoking tobacco, betel quid chewing, human papillomavirus infection, and nutritional deficiencies. Understanding the molecular mechanism of oral cancer is vital. The traditional treatment for patients with oral squamous cell carcinoma (e.g., surgery, radiotherapy, and chemotherapy) and targeted molecular therapy still have numerous shortcomings. In recent years, the use of phytochemical factors to prevent or treat cancer has received increasing attention. These phytochemicals have little or no toxicity against healthy tissues and are thus ideal chemopreventive agents. However, phytochemicals usually have low water solubility, low bioavailability, and insufficient targeting which limit therapeutic use. Numerous studies have investigated the development of phytochemical delivery systems to address these problems. The present article provides an overview of oral cancer including the etiological factors, diagnosis, and traditional therapy. Furthermore, the classification, dietary sources, anticancer bioactivity, delivery system improvements, and molecular mechanisms against oral cancer of phytochemicals are also discussed in this review.

## 1. Introduction

Oral cancer refers to cancers that originate in the mouth. Oral squamous cell carcinoma (OSCC) is the commonest type of oral cancer. It is characterized by aggressive tumors of varying degrees of differentiation that tend to have extensive lymph node metastases at an early stage. Therefore, the overall 5 years survival rate for OSCC was below 50% in the past few decades [1,2]. Traditional treatments of patients with OSCC (e.g., surgery, radiotherapy, and chemotherapy) and targeted molecular therapy still have numerous shortcomings. Therefore, we aimed to identify methods for prevention and treatment that may alter the outcome of this disease. Phytochemicals are natural biologically active compounds with potential health benefits; they have become a focus in recent years. Phytochemicals have been demonstrated to have anticancer effects, mainly by regulating epigenetics/epigenomics [3]; targeting cancer stem cells (CSCs) [4]; inhibiting cancer metastasis [5]; improving human immunity, antioxidation activity and anti-inflammatory responses [6]; inhibiting cancer cell cycle progression [7]; inhibiting cell signal transduction [8]; promoting cancer cell apoptosis [7]; and eventually, achieve the effect of inhibiting cancer cell proliferation and angiogenesis [9,10,11]. Several key mechanisms have been explored in leading to cell cycle arrest, proliferation inhibition, and apoptosis induction, such as down-regulation of cell cycle regulators cyclins [12,13,14] and cyclin dependent kinases [12], PI3K/Akt/mTOR and Erk signaling [15], anti-apoptotic proteins Bcl-2 and Bcl-xL [13], as well as enhancing tumor suppressor p21 [12,13] and p53 [13], pro-apoptotic proteins Bax, caspases and poly (ADP-ribose) polymerase (PARP) [13,14]. The antioxidant defense mechanism of phytochemicals enhances specific genes encoding antioxidant proteins through the key transcription factor Nrf2, and its downstream antioxidant proteins include heme oxygenase-1, catalase, glutathione peroxidase, quinone oxidoreductase 1, superoxide dismutase, glutathione S-transferase, and glutamate cysteine ligase [16,17,18]. Finally, the anti-inflammatory mechanism of phytochemicals is mediated by inhibiting the activity of several pro-inflammatory cytokines, including TNFα, IL-1, IL-6, iNOS, COX-2, and nuclear factor-κB (NFκB) [19]. In this review, we discuss the potential of selected phytochemicals for the prevention and treatment of oral cancer.

## 2. Overview of Oral Cancer: OSCC

### 2.1. Definition of OSCC, Incidence, and Mortality

Oral cancer, a subgroup of head and neck cancer, is one of the most common malignancies globally. The OSCC accounts for over 90% of all oral cancers and 38% of head and neck tumors [20,21], and is defined as a malignant epithelial neoplasm exhibiting squamous differentiation characterized by the formation of keratin or the presence of intercellular bridges [22]. Anatomically, the oral cavity includes several subsites: lips, anterior two-thirds of the oral tongue, floor of the mouth, buccal mucosa, upper and lower gingivae, retromolar trigone, and hard palate [23,24]. The OSCC is usually observed in three major subsites: the tongue, buccal mucosa, and lips [25]. A report by the International Agency for Research on Cancer (IARC) [26] “Global Cancer Statistics 2018” reported that 18.1 million new cancer cases and 9.6 million cancer deaths occurred worldwide in 2018. An estimated 354,864 new cases and 177,384 deaths of men and women of all ages worldwide reported lip and oral cancers [26]. A database provided by a tool that predicts the future cancer incidence and mortality burden worldwide indicated that the number of lips and oral cancers will increase from 354,864 to 545,396, and the number of deaths will increase from 177,384 to 275,164 from 2018 to 2040 [27].

### 2.2. Risk Factors

#### 2.2.1. Tobacco Smoking

The IARC has classified tobacco smoking as a type 1 carcinogen in the oral cavity including smokeless tobacco [28]. Smoking accounts for 75% of all oral cancer cases [29]. Smokers have a two to five times higher risk of developing oral cancer compared with nonsmokers [30]. Numerous clinical and epidemiological data have indicated that OSCC caused by tobacco smoking may cause epigenetic changes in oral epithelial cells, suppress immune system function, and cause oxidative stress [31]. Tobacco smoke contains at least 70 carcinogens and cancer-promoting substances [32]. Examples of such carcinogens are nicotine, hydrogen cyanide, formaldehyde, heavy metals (e.g., lead, arsenic), ammonia, radioactive elements (e.g., uranium), phenol, benzene, carbon monoxide, nitrosamines, and polycyclic aromatic hydrocarbons [33,34]. Among them, tobacco-specific nitrosamines (TSNAs) and polycyclic aromatic hydrocarbons (PAHs) may play critical roles in cancer [33,35]. The TSNAs, such as N’-nitrosonornicotine (NNN) and 4-(methylnitrosamino)-1-(3-pyridyl)-1-butanone (NNK), are involved in the metabolic activation of NNK and NNN, which induce harmful mutations in oncogenes and tumor suppressor genes by forming covalently bound DNA adducts that are considered an onset of carcinogenesis [35,36]. The carcinogen benzo[a]pyrene (BaP) is the most thoroughly studied prototypical PAHs. The BaP is the main component of cigarette smoke. Cytochrome P450 enzymes can metabolize BaP and activate it into a carcinogenic reactive intermediate or metabolite. The produced substances can bind to DNA and form DNA adducts that interfere with DNA replication [37]. Metabolically activated BaP may cause cytotoxicity, teratogenicity, genotoxicity, immunotoxicity, mutagenesis, and carcinogenesis [37,38].

#### 2.2.2. Alcohol

Alcohol consumption has been considered a critical risk factor for the development of oral cancer. Alcohol can cause local changes, including (1) changing the morphology of the oral mucosa and, thus, increasing permeability; (2) dissolving the lipid component of the epithelium and, thus, reducing its thickness and causing atrophy; (3) destroying DNA synthesis and repair through the mutagenic and carcinogenic effects of metabolite acetaldehyde and interfering with the enzymes responsible for DNA repair by binding to proteins; and (4) destroying salivary gland function and, thus, causing a reduction in the local carcinogen clearance rate. These changes increase the risk of oral cancer [39,40,41,42].

#### 2.2.3. Betel Quid

The IARC revealed that betel quid and areca nut are not only psychostimulant and addictive substances but have also been classified as group I carcinogens in humans [43]. The main components of areca nut are betel quid alkaloids and polyphenols which may cause oral and pharynx cancer [44]. Betel quid alkaloids are considered to be the active ingredient of the areca nut, and the arecoline in alkaloids is the main source of toxicity, followed by arecaidine. Arecoline and arecaidine are mutagens that can induce DNA strand breaks, chromosome aberrations, sister chromatid exchange, and micronucleus formation in mammalian cells [45]. Furthermore, arecoline can also form areca nut–specific nitrosamine substances (areca-specific N-nitrosamines) through nitrosation reaction in the oral and digestive tract of the human body, thereby causing abnormal cell proliferation and canceration [46]. The polyphenols contained in areca nut have carcinogenic and anti-carcinogenic effects. For example, some reports indicate that the beneficial effects of polyphenols include antioxidant capacity to prevent damage caused by oxidative stress [47] as well as their biological effects through chromatin remodeling and other epigenetic modifications [48,49,50]. On the contrary, a series of studies have shown that polyphenols are another potential active carcinogen because they produce ROS (e.g., HO) and further form 8-OH-dG when mixed with lime to form oral environment alkalines (pH ≥ 9.5) which subsequently destroys DNA [44,51,52]. Although lime itself is not mutagenic, the interaction between polyphenols and lime is the main determinant of ROS generation [44]. Furthermore, ROS can attack salivary proteins and oral mucosa, cause changes in the structure of oral mucosa, and assist in the penetration of toxic substances [53].

#### 2.2.4. Human Papillomavirus

Currently, over 200 different human papillomavirus (HPV) subtypes have been isolated from humans [54], the most common is the high-risk subtype HPV-16 which is classified by IARC as a cause of oral and pharyngeal tonsil cancers [55]. The less common subtype HPV-18 is also classified as a cause of oral cancer [55]. Evidence indicates that HPV enhances mutagenic effects through the two major virus-encoded oncoproteins E6 and E7. These proteins regulate the cell cycle, apoptosis, and genetic stability signaling pathways by increasing the mutation of tumor suppressor genes *p53* and *RB1* and the degradation of their products, which may cause lesions in human oral epithelial cells [56,57,58].

#### 2.2.5. Nutritional Deficiencies

Insufficient dietary intake of vegetables and fruits causes nutrient and mineral deficiencies (e.g., carotenoids, antioxidant vitamins, phenols, terpenoids, steroids, indoles, and fibers), which increases the risk of cancer. These foods contain protective bioactive compounds called phytochemicals. A lack of phytochemicals is believed to contribute to the development of oral diseases [59,60].

#### 2.2.6. Other Factors

Several studies have demonstrated that the risk of cancer is increased by several other factors such as immune conditions (e.g., congenital defects in the immune system and organ transplant recipients who are administered immunosuppressant drugs), environmental pollutants (e.g., arsenic, chromium and nickel), occupational exposures (e.g., ultraviolet radiation), microorganisms (e.g., bacteria), and genetic diseases (e.g., Fanconi anemia, dyskeratosis congenita, and Bloom syndrome) [61,62,63,64].

### 2.3. Pathological Symptoms

Clinical manifestations and histopathological features are the main basis of clinical diagnosis, and OSCC originates from precancerous lesions of the internal squamous epithelium of the oral cavity [65]. Common signs include leukoplakia, erythroplakia, submucosal fibrosis, verrucous hyperplasia, lichenoid dysplasia, and chronic ulcers in various parts of the oral cavity [66,67,68].

#### 2.3.1. Clinical Manifestations

The most common clinical precancerous lesions of OSCC are hyperplasia or atrophy following chronic inflammation or carcinogenic stimuli, characterized by leukoplakia, erythroplakia, or erythroleukoplakia [61]. The two main types of leukoplakia are homogeneous leukoplakia (generally smooth, uniformly thin and cracked, with consistent whiteness) and nonhomogeneous leukoplakia (generally variable thickness and different shapes such as fissured, granular, nodular, and even verrucous). Nonhomogeneous leukoplakia carries a higher risk of malignant transformation than homogeneous leukoplakia [69,70]. The prevalence of erythroplakia is relatively low; however, it has a higher potential to transform into malignant tumors than leukoplakia [66,71]. Histopathologies have demonstrated that 51% of erythroplakia lesions are invasive SCC, 40% are carcinoma in situ, and 9% are mild or moderate dysplasia [72]. The carcinogenic progress of patients with erythroleukoplakia is nearly four times that of patients with homogeneous leukoplakia [73]. The three clinical forms of OSCC may eventually develop into endophytic necrotizing ulcers with irregular and convex induration borders or develop into exophytic clumps. The surface texture may be verrucous, pebbled, or relatively smooth [74]. Furthermore, malignant OSCC changes may also occur in oral submucosal fibrosis and lichen planus. Oral submucous fibrosis is a chronic inflammation that is associated with fibrous lesions of the oral mucosa. The typical clinical features are a burning sensation of the oral mucosa, dry mouth, blanching, stiffening, and ulceration [75]. Oral lichen planus is a chronic inflammatory autoimmune disease mediated by T cells [68]. The clinical manifestations can be divided into papular, plaque-like, atrophic, erosive, linear, reticular, or annular. Among the clinical manifestations, atrophy, ulcer, and erosion have the highest malignant transformation rates [76].

#### 2.3.2. Histopathological Features

In 2017, the World Health Organization issued a revised diagnosis and grading of oral epithelial dysplasia based on a combination of eight architectural and eight cytological criteria. The architectural changes include irregular epithelial stratification, loss of polarity of the basal cells, drop-shaped rete ridges, increased number of mitotic figures, abnormal superficial mitosis, premature keratinization in single cells (dyskeratosis), keratin pearls within rete ridges, and loss of epithelial cell cohesion. The cytological changes include abnormal variation in nuclear size, abnormal variation in nuclear shape, abnormal variation in cell size, abnormal variation in cell shape, increased nuclear–cytoplasmic ratio, atypical mitotic figures, increased number and size of nucleoli, and hyperchromasia [77]. Mild dysplasia indicates that the change occurs only in the lower third of the epithelium with a slight polymorphism in the cell or nuclei. Moderate dysplasia exhibits atypical cell hyperplasia that extends to the middle third of the epithelium. Cytological changes are characterized by obvious cell and nuclear abnormalities, accompanied by abnormal mitosis in the basal layers. Architectural changes may cause bulbous rete pegs and are often accompanied by hyperkeratosis. In cases of severe dysplasia, abnormal hyperplasia from the basal layer to the upper third of the epithelium is observed. The cytological changes further include enlarged nuclei, increased nucleoli, significant polymorphism, and mitosis. The architecture usually loses its layering completely. Furthermore, severely abnormal keratinization or even the formation of keratin pearls are observed, and abnormal forms of bulbous rete pegs appear [65,77,78]. Carcinomas in situ are the most severe form of epithelial dysplasia, characterized by full-thickness cytological and architectural changes. Microinvasive OSCC is an early malignant tumor that is characterized by infiltration of the superficial lamina propria, usually between 0.5 and 2 mm deep, and is often accompanied by reactive desmoplasia [79,80].

### 2.4. Molecular Mechanism

#### 2.4.1. Acquisition of Autonomous Proliferative Signaling

In normal cells, growth factors bind to specific receptors to stimulate cell proliferation and differentiation. However, cancer cells have autonomous, chaotic growth characteristics because of the dysregulation of growth signals [81]. Studies have established that overexpression of epidermal growth factor receptor (EGFR) is significantly associated with an advanced clinical stage of OSCC, a poor survival rate, and an increased risk of recurrence [82]. One of the most frequently altered signaling pathways in squamous cell carcinoma of the head and neck is the EGFR/PI3K/Akt cascade [83,84]. The PI3K pathway is activated when EGFR binds to ligands such as EGF or transforming growth factor-α (TGF-α), and subsequently promotes phosphatidylinositol 3,4,5-trisphosphate production which initiates Akt activation. Then, Akt activates the NF-κB-mediated antiapoptotic pathway and prevents FOXO-mediated proapoptotic target transcription to promote cell survival [85,86]. Furthermore, cyclin D1 regulates the cell cycle from G1 to S. Cyclin D1 overexpression shortens the G1 phase, resulting in abnormal cell proliferation which may eventually promote the occurrence of other genetic damage. Studies have demonstrated that the expression of cyclin D1 is related to the degree of differentiation, lymph node metastasis, and a poor OSCC prognosis [87,88]. *Ras* is one of the most common mutant oncogenes in oral cancer [89,90]. Point mutations in the *Ras* gene can cause the Ras protein to be permanently activated and induce continuous cell growth. Furthermore, the family of signal transducer and activator of transcription proteins plays a critical role in the development of oral cancer. A study demonstrated that STAT3 promotes OSCC cell migration, invasion, epithelial–mesenchymal transition (EMT), and aerobic glycolysis by inhibiting FOXO1 transcription [91]. Furthermore, a study demonstrated that the expression of phosphorylated STAT3 is related to poor OSCC prognosis [92].

#### 2.4.2. Evasion of Growth Suppressors

Several tumor suppressor genes in cancer cells lose their function through mutation, deletion, and methylation [93]. Mutation of the tumor suppressor gene *p53* occurs in almost all human tumors. Furthermore, loss of heterozygosity (LOH) at the *p53* locus is detected in 47–66% of head and neck cancers and 35% of early oral dysplasia [93,94]. Another mechanism for promoting p53 degradation is by HPV infection through interaction with the viral regulatory protein E6 to cause ubiquitination and proteolysis of p53 [95]. The tumor suppressor p16^INK4A^ is a cell cycle inhibitor. Hypermethylation of the *p16^INK4A^* promoter causes gene silencing which is common in precancerous oral lesions [96].

#### 2.4.3. Evasion of Apoptosis

The Bcl-2 gene family and related proteins form the core and main effector of the apoptosis program, especially antiapoptotic proteins Bcl-2 and Bcl-xL and proapoptotic proteins Bax and Bak. Coutinho-Camillo et al. [97] demonstrated that Bcl-2 family proteins are involved in OSCC tumorigenesis, differentiation, and metastasis and are associated with poor prognosis.

#### 2.4.4. Immortalization

Each replication cycle results in the loss of a small amount of telomeric DNA used as a protective cap. When sufficient loss occurs, cell death is induced. Telomerase prolongs telomeres, allowing cells to replicate indefinitely during immortalization. Telomerase activity is upregulated in both oral epithelial dysplasia and OSCC cases [98].

#### 2.4.5. Induction of Angiogenesis

Growing tumors require an increased blood supply to obtain sufficient oxygen and nutrients. Therefore, a rich blood vessel network can cause tumors to reach a clinically obvious size and acquire metastatic ability. Vascular endothelial growth factor (VEGF) is an effective stimulator of blood vessel formation, and VEGF expression was determined to increase in all stages of tumor progression. Other critical factors include fibroblast growth factor, platelet-derived growth factor, TGF-β, TNF-α, interleukin 8, and angiopoietins [99]. In OSCC, the microvessel and lymphovessel densities are closely related to tumor size, clinical stage, lymph node metastasis, recurrence rate, and prognosis [100].

#### 2.4.6. Tissue Invasion and Metastasis

The critical steps for cancer cell invasion and metastasis are to reduce the adhesion and isolation of tumor cells, the proteolysis of the extracellular matrix, and the migration of tumor cells. The downregulation of E-cadherin reduces the strength of cell adhesion in tissues, resulting in tumor cells easily invading surrounding tissues through the basement membrane. In OSCC, the reduction of E-cadherin is related to invasion, lymph node metastasis, and poor prognosis and can be used as a biomarker to evaluate the progress and prognosis of oral dysplasia and OSCC [101,102]. The degradation of the extracellular matrix and basement membrane requires the participation of matrix metallopeptidases (MMPs). Numerous studies have described the relationship between MMPs and OSCC, which is primarily involved in tumor progression, invasion and metastasis, and poor prognosis [103,104,105]. Integrins are cell adhesion molecules that mediate the interaction between cells and the extracellular matrix and regulate a variety of functions, including cell motility, invasion, proliferation, and migration [106,107].

### 2.5. OSCC Diagnosis

#### 2.5.1. Tumor, Node, Metastasis (TNM) Staging System

The tumor, node, metastasis (TNM) staging system is one tool to classify OSCC, and is based on the assessment of the size of the primary tumor (T), the extent of regional lymph node involvement (N), and the presence of distant metastases (M) [108,109]. In the eighth edition of the AJCC TNM staging manual, the depth of invasion of the primary tumor and the extranodal expansion of lymph node metastases have been included in the T and N pathological categories, respectively [110,111]. However, approximately 20–40% of patients with OSCC have no clinical or imaging evidence of metastasis to lymph nodes; however, histopathological examination of local lymph nodes revealed metastatic growth [112]. Therefore, TNM staging alone is usually insufficient to diagnose the factors critical to OSCC survival. Assessing other characteristics of the tumor, such as the degree of differentiation and the type of invasion, can help in terms of obtaining accurate diagnoses, selecting favorable treatment methods and reliably evaluating the management results.

#### 2.5.2. Imaging Evaluation

Because early OSCC usually involves superficial lesions, using medical imaging, such as computed tomography (CT), magnetic resonance imaging (MRI), and positron emission tomography (PET), is crucial for diagnosing and determining the scope of the primary lesion, local lymph node spread, and distant metastasis [61]. Among them, CT can provide a specific diagnosis for the areas of matrix mineralization, lesion density and pattern, and extent of adjacent bone and neurovascular involvement [113]. In addition, MRI is complementary to CT for evaluating head and neck pathology, and provides a clearer contrast resolution than CT for evaluating the extent of local soft-tissue infiltration, medullary bone involvement, and perineural invasion. [114,115]. Furthermore, PET scans can provide physiological and biochemical data related to cell metabolism by using intravenous injection of biological radioactive chemicals that have different biological substance features. These phenomena usually occur before tissue structure changes; thus, PET images may reveal abnormalities before CT or MRI [116]. However, the inability to clearly detect positioning is a critical drawback of the PET method. To overcome this problem, anatomically superior imaging methods (e.g., CT and MRI) are used in conjunction with PET (i.e., PET–CT and PET–MRI).

#### 2.5.3. Biopsy

Although noninvasive imaging techniques can be used to characterize head and neck tumors, tissue sampling is still the reference standard for the definitive diagnosis of soft-tissue tumors. Exfoliative cytology is a minimally invasive technique. The desquamated or abraded cells can be collected for microscopic examination, and the cytomorphometric changes are assessed as a reliable indicator of dysplasia or neoplastic changes [117,118]. Incisional and excisional biopsies are the most commonly used techniques. An incisional biopsy removes a small piece of a suspicious tissue sample from the lesion or mass for diagnosis, whereas excisional biopsy removes the entire tumor and some surrounding normal tissue. Fine needle aspiration biopsy is the use of a thin, hollow needle connected to a syringe to draw a small amount of liquid and very small tissue blocks from a tumor and then observe the presence of tumor cells under a microscope [119]. Liquid biopsy is a noninvasive diagnostic tool that assesses blood, saliva, or other body fluid samples (e.g., urine, seminal plasma, pleural effusion, cerebrospinal fluid, sputum, and feces) [120].

#### 2.5.4. Biomarkers

Biomarkers refer to “biomolecules identified in blood, other body fluids, or tissues that indicate normal or abnormal processes, conditions, or diseases, and can be used to observe the body’s response to treatments for diseases or conditions” [121]. Studies have investigated the use of nucleic acid and protein changes in saliva, blood, and tissue samples as biomarkers to improve the diagnosis and prognosis of OSCC [122]. Numerous studies have demonstrated that the loss of specific chromosomal regions in known or speculated tumor suppressor genes can be used as an early predictor of the subsequent progression of oral precancerous lesions [123]. Abnormal DNA methylation can silence tumor suppressor gene expression, which is a common phenomenon in malignant tumors and leads to tumorigenesis, aggressiveness, invasiveness, and malignant transformation of oral epithelial dysplasia [124]. MicroRNAs (miRNAs) are functional noncoding RNAs that are involved in posttranscriptional gene regulation; miRNA can regulate the expression of target genes involved in cancer biology by acting as oncogenes or tumor suppressor genes [125]. Therefore, the deregulation of specific miRNAs can engender the initiation and progression of cancer; thus, they are attractive candidates as biomarkers of oral tumors. Furthermore, quantitative proteomics technology has analyzed potential biomarkers for local and systemic diseases, which could help identify specific proteins involved in disease development and quickly diagnose diseases [126].

### 2.6. Therapeutic Alternatives

Numerous treatments are available for OSCC, depending on the size, location, and stage of the tumor and the overall health of the patient. For patients with stage 1 and stage 2 oral cancer without lymph node spread, no clinical or radiological evidence indicates that metastasis, radiotherapy, or surgical resection is the preferred treatment. Patients with stage 3 and stage 4 (advanced) cancer or with high-risk characteristics should increase the combined treatment of postoperative radiation and chemotherapy. However, the treatment effect of these traditional methods is limited. Targeted molecular therapies have been applied to patients with oral cancer to reduce the side effects of nonspecific cell death and, thus, improve survival rates.

#### 2.6.1. Surgical Resection

For resectable tumors, surgery is superior to all other therapies, but for surgical resection, sufficient margins for the primary tumor must be available. Failure to obtain a clear surgical margin leads to an increased risk of local recurrence and subsequently a poor prognosis [127].

#### 2.6.2. Radiation Therapy

The radiotherapy of OSCC can be achieved by using external beam radiotherapy and brachytherapy [128]. External beam radiotherapy employs special instruments (e.g., linear accelerators and betatrons) to generate high-energy radiation (e.g., X-rays) which can destroy cell chromosomes and prevent cell proliferation when focused on the cancer site from the outside, thereby eliminating cancer cells that can divide and grow rapidly. Brachytherapy implants traditionally provide high-dose radiation to limited tissues through the insertion of an iridium-192 needle or iodine-125 seed, which can retain the surrounding normal tissues [129,130]. Radiation therapy is often used in conjunction with surgery or chemotherapy to kill mitotic cells by destroying DNA.

#### 2.6.3. Chemotherapy

Chemotherapy can be used to treat cancers that cannot be surgically removed because of size or dispersion. Chemotherapy slows the growth of cancer for as long as possible and helps relieve any symptoms caused by cancer. The most commonly used chemical drugs for oral cancer are cisplatin, carboplatin, 5-fluorouracil, paclitaxel, docetaxel, and hydroxyurea, which can be used alone or in combination with other drugs [131,132,133].

#### 2.6.4. Targeted Therapy

The EGFR antagonist cetuximab monoclonal antibody is the only first-line targeted therapy for OSCC; it was approved by the U.S. Food and Drug Administration (FDA) in 2006. Cetuximab can inhibit the binding of EGF and EGFR, which interferes with the downstream response induced by EGFR activation. Furthermore, cetuximab enhances local tumor control using radiation therapy [134]. However, patients have a high probability of developing drug resistance in the later period of cetuximab which hinders the clinical treatment effect [135].

#### 2.6.5. Immunotherapy

Immunotherapy has been developed as an effective cancer treatment, which controls and kills tumor cells by reducing the suppression state of immune cells in the tumor microenvironment and activating the body’s immune function. Tumors employ certain immune-checkpoint pathways as the main mechanism of immune resistance, especially against T cells that target tumor antigens. Programmed cell death-1 (PD-1) is an immune checkpoint on the surface of T cells. When T cells are activated, PD-1 expression is induced. However, when PD-1 binds to its ligand PD-L1 on tumor cells, it can block the PD-1 pathway and suppress the immune response of T cells, thereby protecting tumor cells from apoptosis caused by autoimmunity [136]. Nivolumab and pembrolizumab are the first two anti-PD-1 mAbs approved by the FDA [137]. All organ systems can be affected by immune-related adverse events of immune-checkpoint inhibitors. In clinical trials, the most commonly reported effects are dermatologic, gastrointestinal, endocrine, respiratory, and hepatic [138].

## 3. Overview of Phytochemicals

Considerable nutritional epidemiological evidence has indicated that regular consumption of fruits and vegetables is linked to a lower risk of cancer [139,140,141,142]. Nutritional compounds have a long history of preventing and treating cancer [142]. Numerous reports have indicated that natural foods, including cruciferous vegetables (e.g., cabbage and broccoli), alliums (e.g., garlic and onion), green tea, citrus fruits, soybeans, tomatoes, berries, and ginger have chemopreventive activity [143]. Therefore, preventing and treating cancer using phytochemicals in natural foods has aroused interest in the academic community. Phytochemicals are derived from plants and are a class of molecules with biological activity. Over 10,000 phytochemicals have been identified worldwide [144,145]. Furthermore, genistein (from soybeans and soy products) [146,147,148], lycopene (such as from tomatoes, red carrots, watermelons, grapefruits, and papayas) [149,150,151], brassinin (from cruciferous vegetables) [152,153,154], sulforaphane (from asparagus) [155,156,157], indole-3-carbinol (from broccoli) [158,159,160], and resveratrol (from grapes, peanuts, peanut butter, red wines) [161,162,163,164,165] are being studied in preclinical or clinical trials for cancer chemoprevention.

### 3.1. Classification and Dietary Sources of Phytochemicals

Phytochemicals can be divided into phenolic compounds, carotenoids, and others. Phenolic compounds contain one (phenolic acids) or more (polyphenols) aromatic rings with attached hydroxyl groups in their structures. Phenolic acid can be classified into hydroxycinnamic acid and hydroxybenzoic acid. Hydroxycinnamic acid is found in cinnamon, coffee, blueberries, kiwis, plums, apples, and cherries. However, hydroxybenzoic acid is found in few consumable plants [49]. Phenolic compounds can be sub-grouped as flavonoids and non-flavonoids [166]. More than 8000 phenolic compounds from plants have been reported, and the half of phenolic compounds is flavonoids presenting as aglycone, glycosides, and methylated derivatives [167]. Flavonoids have several subgroups which include flavonols, flavones, flavanonols, flavanols, flavanones, anthocyanidins, chalcones, and isoflavones [5,168]. Non-flavonoids compounds include phenolic acids, tannins, coumarins, lignans, and stilbens [169]. Among the phenolic compounds, isoflavones, stilbene, coumestan, and lignan have estrogenic activity, so they have been denominated as phytoestrogen [170,171]. Among them, coumestans are produced by oxidation of isoflavones-derived pterocarpan; while stilbene and lignan are non-flavonoids group [172]. Phytoestrogens are natural substances that can exert estrogen-like activity because of their chemical structure which is similar to human estrogens (17-β-estradiol). The commonest phytoestrogens in the diet are isoflavones and lignans, and their main food sources are legumes (especially soybeans) [170]. The affinity of phytoestrogens to estrogen receptors (ERs) is 1/100 to 1/10,000 that of 17-β-estradiol. However, phytoestrogens may reach micromolar concentrations in the bloodstream [170] and, thus, act as both agonists and antagonists. The ER-α and ER-β are two distinct ERs. The ER-β is a modulator of ER-α activity. Phytoestrogens are defined as selective estrogen receptor modulators and display a higher binding affinity with ER-β compared with ER-α [173]. For example, the binding affinity of isoflavone genistein with ER-β is 20 to 30 times higher than with ER-α [174], which explains why the administration of phytoestrogens does not produce the classic side effects associated with estrogen administration (cerebrovascular and cardiovascular attacks, higher incidences of endometrial, and breast cancer).

Carotenoids are members of the tetraterpenes family that are responsible for the yellow, orange, or red color of fruits, leaves, and flowers. Carotenoids occur in all organisms capable of photosynthesis, the conversion of solar energy into chemical energy. In plants, carotenoids contribute to photosynthetic machinery and protect plants from photodamage. Nearly 600 carotenoids have been identified in nature. However, only approximately 50 carotenoids are identified in a typical human diet, and approximately 20 carotenoids are present in human blood and tissues [175]. Carotenoids can be divided into provitamin A (e.g., α-carotene, β-carotene,γ -carotene, and β-cryptoxanthin) and non-provitamin A compounds [175]. Carotenoids are also classified into two groups, namely, carotenes and xanthophylls, based on their chemical constituents. Carotenoids composed only of hydrocarbons are carotenes, including lycopene, α-carotene, β-carotene, and their oxygenated derivatives are xanthophylls, including β-citraurin, which contains an aldehyde group; neoxanthin, antheraxanthin, and violaxanthin, which contain epoxide groups; canthaxanthin and echinenone, which contain oxo/keto groups; and β-cryptoxanthin, zeaxanthin, and lutein, which contain hydroxyl groups [176]. The most common dietary carotenoids are three carotenes (α-carotene, β-carotene, and lycopene) and three xanthophylls (β-cryptoxanthin, lutein, and zeaxanthin) [144]. Dietary carotenoids have been hypothesized to reduce the risk of cancer because of their antioxidant function. Epidemiological studies have demonstrated a correlation between a high carotenoid intake in the diet and a low risk of cancer [177]. The intake of carotenoids potently inhibits cell proliferation, arrests the cell cycle in different phases, and increases apoptosis and antioxidants in cancer cells [175]. β-Cryptoxanthin (a carotenoid) and hesperidin (a flavonoid) display anticancer effects in several tissues. Gavage with pulp or juice containing high levels of β-cryptoxanthin and hesperidin can inhibit chemically induced rat colon, rat tongue, and mouse lung cancer [178].

**Figure 1:**
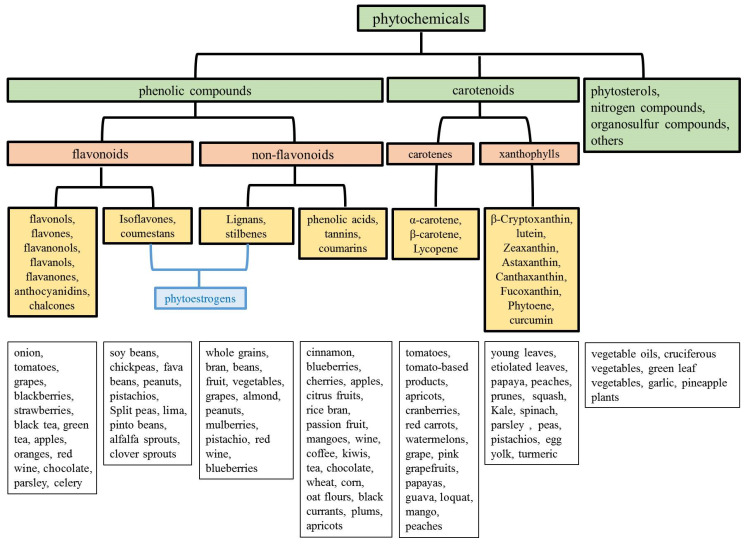
Classification and dietary sources of phytochemicals.

In addition to phenolic compounds and carotenoids, phytosterols, nitrogen compounds, and organosulfur compounds are also commonly discussed phytochemicals. Phytosterols are plant derived lipid compounds and usually found in plants or macro fungi, which resemble cholesterol. They can be further classified as sterols, (unsaturated compounds) and stanols (saturated molecules) [169]. Nitrogen compounds are a subclass that contain nitrogen atoms and are classified as alkaloids (a group of plant secondary metabolites, biosynthesized from amino acids) and non-alkaloid derivatives (such as protoalkaloids, pseudoalkaloids, alkamides, lectines, cyanogenic glycosides) [169]. Organic sulfur compounds are a subclass that contain sulfur atoms and mainly found in garlic and cruciferous vegetables [179]. The classification and dietary sources of common phytochemicals are listed in Figure 1.

### 3.2. Anticancer Bioactivity of Phytochemicals

Overproduction of oxidants (reactive oxygen species and reactive nitrogen species) in the human body is responsible for the pathogenesis of cancer. Phytochemicals present in fruits, vegetables, and grains have a protective effect against the development of cancers. The protective role of phytochemicals may be associated with their antioxidant activity. Studies have suggested that lifestyle changes could prevent more than two-thirds of human cancers and that dietary factors contribute to approximately 35% of human cancer mortality [145]. Free radicals are thought to be related to multistage carcinogenic processes. Peroxyl radicals and lipid peroxidation can independently cause DNA mutations, which are essential for the initiation of the carcinogenic process. Antioxidant phytochemicals can regulate the initiation of carcinogenic processes by protecting against DNA damage. For example, green tea polyphenols, silymarin from milk thistle, and proanthocyanidins from grape seeds could protect the skin from the adverse effects of UV radiation (e.g., the risk of skin cancers) through four principal mechanisms: reducing UV radiation-induced inflammation, oxidative stress, DNA damage, and immune responses. Furthermore, phytochemicals could inhibit cell proliferation and induce cancer cell death. Quercetin, genistein, and resveratrol exhibited higher induction of quinone reductase. The upregulation of quinone reductase is thought to be a useful biomarker for anticarcinogenesis [145]. Furthermore, genistein displayed anticancer effects on breast cancer by demethylating and reactivating methylation-silenced tumor suppressor genes [180]. Lycopene and β-carotene could inhibit cell proliferation, arrest the cell cycle, and increase apoptosis of human breast cancer cells [181]. In conclusion, oxidative stress contributes to all phases of tumorigenesis (i.e., initiation, promotion, and progression) either by using a direct mechanism involving DNA damage or indirectly by regulating cell signal transduction. Therefore, the reduction of oxidative stress plays a key role in chemoprevention. Dietary phytochemicals have broad prospects through their antioxidant properties. Metastasis represents a serious complication in cancer treatment. Studies have shown flavonoids have anti-cancer effects by regulating key signaling pathways involved in critical steps of metastatic spread in several in vitro and in vivo models [5,182]. Epigenetic changes could occur in the early stages of carcinogenesis preceding genetic mutations, so epigenetics are considered to be promising targets for early interventions against cancer using epigenetic biologically active substances [3]. Several phytochemicals (e.g., curcumin, quercetin, apigenin, (−)-epigallocatechin-3-gallate (EGCG), genistein, resveratrol, sulforaphane, and diallyl disulfide) exert significant chemopreventive effects by targeting multiple anticancer pathways as well as epigenetic mechanisms [3,183,184]. The CSCs are a rare subpopulation of cancer cells with abnormal regulation of self-renewal, proliferation or apoptosis, which lead to cancer progression, invasiveness, metastasis formation, and chemotherapy resistance [4]. In various studies, EGCG [185,186], resveratrol [187,188], genistein [189], curcumin [190,191], sulforaphane [192], and diallyl trisulfide [193] have targeted CSCs to express its anticancer ability [4]. Furthermore, phytochemicals exhibit little or no toxicity to healthy tissue and, thus, could be ideal chemopreventive agents.

### 3.3. Bioavailability and Delivery System Improvements of Phytochemicals

The therapeutic effects of natural compounds are usually limited by low water solubility, low bioavailability, and deficient targeting. In order to solve these problems, several studies have focused on the developments of phytochemical delivery systems (e.g., liposomes, nanoparticles, nanoemulsions, films, adjuvants, micelles, and phospholipid complexes) [194]. These delivery systems would enable flavonoids, resveratrol, celastrol, curcumin, berberine, and camptothecin to avoid drug metabolism, overcome physiological barriers, and achieve delivery at a higher concentration at cancer sites [195]. For example, resveratrol has a circulation half-life of several minutes, and flavonoids quercetin and EGCG usually have low micromolar concentrations in the blood, which can be sufficient for cytoprotective action but are considered insufficient to achieve anticancer effects [195,196,197]. Different delivery systems for resveratrol have been developed (e.g., the resveratrol encapsulation in lipid nanocarriers or liposomes, emulsions, micelles, insertion into polymeric nanoparticles, solid dispersions, and nanocrystals). These systems can facilitate the rapid absorption of large quantities of resveratrol, which then effectively increases plasma concentrations [198]. Furthermore, nanoencapsulation of polyphenols (EGCG, quercetin, curcumin and resveratrol) could prolong circulation, improve localization, enhance efficacy, and reduce the risk of multidrug resistance [199].

## 4. Molecular Mechanisms of Phytochemicals against Oral Cancer

The continuous increase in cancer cases, the failure of conventional chemotherapies, and the excessive toxicity of chemotherapies demand alternative cancer treatments [200]. Phytochemicals can inhibit or antagonize factors, which are dysregulated in cancer cells and may enhance the effects of conventional therapy or could be developed into a stand-alone therapy [140]. Because of the historical presence of phytochemicals in the human diet, the risk of severe adverse events may be lower in therapeutic settings compared with synthetic compounds that are entering the human body for the first time. Phytochemicals may exert their chemopreventive properties by blocking the critical events of tumor initiation and promotion, thereby reversing the premalignant stage. Phytochemicals may also prevent tumorigenesis by inhibiting or slowing tumor progression or promoting cell differentiation. Furthermore, phytochemicals can enhance innate immune surveillance and improve the elimination of transformed cells [201]. In this review, we describe the molecular mechanisms of certain phytochemicals, including black raspberries (BRBs), green tea, EGCG, (−)-epigallocatechin (EGC), (−)-epicatechin-3-gallate (ECG), resveratrol, lycopene, curcumin, garlic, onion, astaxanthin, canthaxanthin, and bromelain for oral cancer chemoprevention and treatment (Table 1).

### 4.1. BRBs

The BRBs are rich in vitamins, minerals, fiber, anthocyanins, phenolic components, and other bioactive components with cancer inhibitory capacities [202], and have been reported to inhibit various cancers including oral cancer [203]. In 2002, in a DMBA-induced hamster cheek pouch model, dietary blackberries were determined to have the potential to inhibit oral cancer formation [204]. The mechanisms associated with the inhibition of oral cancer by BRBs were immediately investigated. In *lacI* rat oral epithelial cells, the extracts of BRB enhances the removal of DNA damage caused by DBP-diol, a primary metabolite of the tobacco-smoke carcinogen, dibenzo-[*a,l*]-pyrene (DBP) [205,206]. In 4-nitroquinoline 1-oxide (4NQO) F344 rats model of oral cancer, BRBs can inhibit oral carcinogenesis by modulating proinflammatory [207,208], apoptotic [208], glycolytic [203], and AMP-activated protein kinase pathways [203]. The local treatment of BRB gel (0.5 g, four times daily) on oral premalignant lesions (OPLs) for 3 months resulted in considerable reductions in lesion size, histologic grade, and LOH at tumor suppressor gene loci [203,209,210].

### 4.2. Green Tea, EGCG, EGC, and ECG

Green tea contains rich flavonoids and other polyphenolic antioxidants, which protect against cancer. Three major constituents of green tea are EGCG, ECG, and EGC. These compounds induced considerable dose-dependent inhibition of cell growth [211]. Among them, EGCG is the major polyphenol to inhibit growth and interfere with the carcinogenic process of various cancer cell lines [212]. Both observational and intervention studies have indicated that green tea intake has a protective effect on the development of oral–digestive tract cancer. Furthermore, oral supplementation of green tea extract had an inhibitory effect on a precancerous lesion of the oral cavity [213]. Studies have indicated that EGCG inhibits both cell proliferation and migration of oral cancer cells, which is associated with a reduction in the expression of phosphorylated EGFR [212], and EGCG can induce cell apoptosis and cell cycle arrest, modulate transcription factors (NF-κB and AP-1), and reduce cell migration and invasion in OSCC cell lines. In animal models of oral carcinogenesis, tea polyphenols reduced oxidative stress and phase I enzymes (cytochrome b5, cytochrome P450, cytochrome b5 reductase, cytochrome P450 reductase, aryl hydrocarbon hydroxylase, and DT-diaphorase) and induced phase II enzyme (glutathione-S-transferase and UDP glucuronyl transferase) [141]. In a mouse xenograft model, EGCG was efficient in inhibiting phorbol-12-myristate-induced cell invasion, MMP-9 expression, and tumor growth [214]. In clinical trials, tea extract intake reduced lesion size in patients with leukoplakia [215]. Tea extract intake reduced stromal VEGF and cyclin D1 expressions in patients with a high-risk of developing OPLs [216]. Clinical trials have also shown that drinking more than 10 cups of green tea per day reduces the risk and delays the onset of cancer compared to those who drink less than 3 cups per day. In addition, smokers taking green tea extract (2000–2500 mg/day) for 4 weeks reduced the DNA damage of oral keratinocytes [217,218].

### 4.3. Curcumin

Curcumin, a xanthophyll carotenoid, is the primary active constituent of turmeric which is derived from the rhizome (root) of *Curcuma longa*. Turmeric is one of the components of curry [219]. The anticancer effects of curcumin and its underlying mechanisms have been studied in several tumor systems including skin, colon, lung, duodenal, stomach, esophageal, and oral cancer. Curcumin inhibits the invasive ability and EMT by reducing the expression of MMP-2 and MMP-9 and modulating the p53-E-cadherin pathway in SCC-25 cells [220]. Curcumin also inhibits cell proliferation of SCC-9 cells by increasing miR-9 expression and inhibiting Wnt/β-catenin signaling [221]. Moreover, curcumin activates p38, which activates the C/EBPα transactivator by interacting with the binding element of the IGFBP-5 promoter in SAS cells [222]. Curcumin can also reduce the tumor burden and tumor incidence in 4NQO-, DMBA-, or MAOMN-induced oral cancer models [223,224]. Local treatment with curcumin provided significant symptomatic relief in patients with external cancerous lesions. A study conducted on 62 patients receiving curcumin treatment reported that 10% of patients had a reduction in lesion size and pain [225]. Curcumin is considered to be pharmacologically safe. The safety and tolerability of curcumin administered at a high dose of 8 g/day were apparent in clinical trials [194,226].

### 4.4. Garlic and Onion

Several studies have indicated that increased consumption of allium vegetables, such as garlic and onion, can reduce the risk of cancer [227]. S-allylcysteine (SAC) is a garlic constituent that has been reported to effectively inhibit cell proliferation and EMT in human oral squamous cancer CAL-27 cells [228,229] and a mouse xenograft model [228]. The administration of SAC significantly inhibits the development of DMBA-induced hamster buccal pouch (HBP) carcinogenesis by modulating lipid peroxidation and enhancing antioxidant activities [230,231]. Furthermore, the combined administration of tomato and garlic significantly inhibits the development of DMBA-induced HBP carcinogenesis by inducing the apoptotic pathway [232]. Although the clinical evidence for the effective doses of garlic for the prevention and treatment of oral cancer is quite limited, no symptoms of garlic toxicity were reported in the literature. According to the clinical trials, increasing the garlic consumption of 20 g/day reduced the risk of gastric and colorectal cancer [233]. Onion extract significantly delayed tumor formation in DMBA-induced HBP carcinogenesis [234,235]. Quercetin, a principal flavonoid compound in onions, induced cytotoxic effects and reduced migration and invasion of SAS cells [236]. Doses of quercetin ranged from 250 to 5000 mg/day and were evaluated safely in 30 untreated patients with chronic hepatitis c virus infection. Quercetin displayed safety, well tolerated, and no adverse events or signs of toxicity in all trial participants [237]. In some countries, quercetin is available as a dietary supplement, and the recommended dose is 200–1200 mg/day [238,239].

### 4.5. Resveratrol

Resveratrol (3,4’,5-trihydroxy-trans-stilbene) is a phytochemical that is naturally produced by numerous plants such as grapes, peanuts, and mulberries. The anticancer properties of resveratrol have been confirmed in various types of cancer including oral cancer [240]. Resveratrol has been shown to inhibit cell growth and DNA synthesis in SCC-25 cell lines. The combination of resveratrol and quercetin can enhance the inhibitory effect of quercetin on cell growth and DNA synthesis [241]. Resveratrol demonstrated considerable efficacy against the growth and proliferation of FaDu and Cal27 cells by inducing DNA damage and apoptosis. The same effect was observed in FaDu tumor xenografts in athymic nude mice [242]. A substantial body of evidence indicates that proinflammatory mediators are associated with cancer progression, invasion, and metastasis. Resveratrol can inhibit or interfere with the main molecular targets of inflammation (e.g., NF-κB and TGFβ), which inhibit the activity of regulatory T (Treg) cells [CD4(+)CD25(+)FoxP3(+)], thereby reducing the risk of cancer [243,244]. Resveratrol has been found to be safe and reasonably well-tolerated at up to 5 g/day single dose or as part of a multiple-day dosing regimen in healthy populations [245,246]. However, choosing the right dose of resveratrol to target a specific disease may be tricky. For example, patients with multiple myeloma taking 5 g of resveratrol (alone or in combination with brtezomib) per day demonstrated renal toxicity, however, no renal toxicity was observed in patients with type 2 diabetics, mitochondrial encephalomyopathy, lactic acidosis, and stroke-like episodes (MELAS) syndrome [247]. For different indications, well-designed human clinical studies are still needed to determine the optimal dose of resveratrol [246].

### 4.6. Lycopene

Lycopene is a fat-soluble carotenoid. The main sources of lycopene in the human diet are tomatoes, tomato-based products, apricots, cranberries, grapes, pink grapefruits, guavas, papayas, peaches, and watermelons. Lycopene has a high singlet oxygen quenching ability. It is a useful food coloring agent because of its strong color and nontoxicity. Furthermore, lycopene plays a multifunctional role in the treatment of oral diseases, such as leukoplakia, oral submucous fibrosis, lichen planus, and OSCC [248]. Studies have evaluated the effects of lycopene in the treatment of oral leukoplakia. The results have demonstrated that patients receiving lycopene had a significant difference in responses compared with a placebo. The observed effect of lycopene indicated that it can be used effectively and safely for the treatment of oral leukoplakia [249]. Lycopene exhibits a strong dose-dependent inhibition of proliferation and promotion of the expression of the gap-junction protein connexin-43in KB1 human oral tumor cells [250]. Furthermore, lycopene administration inhibited oral cancer by modulating lipid peroxidation and enhancing the activities of the enzymes in the glutathione redox cycle in a DMBA-induced oral cancer model [251]. The formulation of lycopene is well tolerated and has minimal side effects. However, the absolute absorption of lycopene does not vary greatly with dose. A significant increase in absolute absorption rate was observed only in the low dose range of 10–30 mg lycopene. One study indicated that there was no significant difference in the amount of lycopene absorbed between men who drank juice containing 120 mg of lycopene and men who drank juice containing 10 mg of lycopene [252]. The results of several independent clinical trial studies have pointed out that lycopene consumption of 10–30 mg/day taken for a period of time can effectively reduce the biomarker levels of cancer in patients with prostate cancer and colorectal cancer [253].

### 4.7. Xanthophylls (Astaxanthin and Canthaxanthin)

Micozzi et al. reported that the concentration of oxygenated carotenoids (xanthophylls) in green leafy vegetables is higher than the α-carotene or β-carotene concentrations (10–20% of total carotenoids), which indicates that xanthophylls may be chemopreventive. The antitumor effects of some xanthophylls, such as canthaxanthin, fucoxanthin, and phytoene, in skin [254], duodenal [255], or mammary tumors [256] have been also reported in limited animal models and in vitro studies. In a 4NQO-induced oral cancer model, the chemopreventive effects of astaxanthin and canthaxanthin (xanthophylls) were investigated. The incidences of tumors and precancerous lesions in rats treated with 4-NQO and astaxanthin or canthaxanthin were significantly lower than in rats treated with 4NQO alone [257]. A clinical study to investigate the antioxidant and anti-inflammatory effects of astaxanthin indicated that the level of C-reactive protein in the blood decreased after taking 2 mg/day of astaxanthin for 8 weeks, indicating that the compound had anti-inflammatory activity [258]. Although many studies have investigated the absolute absorption rate and effective concentration of xanthophylls in animal models, but human clinical trials are rarely implemented. Future studies will need to focus on human clinical trials.

### 4.8. Bromelain

Bromelain is a cysteine protease that is mainly extracted from pineapple plants. The anti-inflammatory and anticancer activities of bromelain are recognized. Bromelain is a complex of thiol endopeptidases and non-protease components including phosphatases, glycosidases, peroxidases, cellulases, glycoproteins, ribonuclease, and carbohydrates. Although this enzyme complex has been widely used in various fields, including medicine, health, food, and cosmetics [259], the complete molecular mechanism of action of bromelain has not been completely identified. However, bromelain gained general acceptability as a phytotherapeutic agent because of its history of safe use and lack of side effects [260]. Bromelain treatment inhibited cell growth and proliferation and induced apoptosis in Ca9-22 and SCC25 human oral squamous carcinoma cell lines through various pathways and G1 cell cycle arrest [261]. Bromelain tends to act as a digestive enzyme and its therapeutic effect may be diminished if taken with food. Therefore, it is recommended to take the bromelain capsule 2 h after the meals. The effective dose of bromelain is 750–1000 mg/day taken for a period of time [262].

## 5. Conclusions

Tumors can be treated by radiotherapy or surgery in the early stages. However, most patients are diagnosed in the later stages of the disease. Therapy outcomes in the later stages have not dramatically improved in recent years. Epidemiological evidence indicates that regular consumption of fruits and vegetables is linked to a lower risk of cancer. Studies have also provided evidence that dietary phytochemicals exert anticancer effects by modulating various molecular mechanisms. Natural compounds are often hindered by low water solubility, low bioavailability, and deficient targeting; thus, numerous phytochemical delivery systems have been developed to compensate for these problems. The encapsulation of EGCG in polylactic acid–polyethylene glycol (PLA–PEG) nanoparticles can enhance the pro-apoptotic and anti-angiogenic potential [272]. In the mammary tumorigenesis model, the silastic implants delivery of ellagic acid requires only 130 times less than the diet route (500 ppm) to achieve similar anti-cancer effects [273]. Compared with the original curcumin conjugated with phosphatidylcholine can increase the bioavailability by five times and significantly reduce the expression of MMP-9 and lung metastasis in the xenograft model of mammary gland tumor [274]. Cancer is caused by multiple factors, so the use of systemic delivery approaches to deliver multiple agents identified as additively or synergistically targeting multiple and overlapping pathways may be more effective than the use of a single drug. The feasibility of using multiple implants to deliver multiple different compounds (curcumin, green tea polyphenols, punicalagin, and diindolylmethane) to a single animal has also been proven [275]. Therefore, if the target organs for the bioaccumulation of chemopreventive agents are identified and delivery systems are developed to increase stability and half-life, great progress can be made in the field of preclinical chemoprevention. In vitro and in vivo experiments on the combined effects of apigenin, curcumin, genistein, resveratrol, EGCG, and sulforaphane with other polyphenols and anticancer drugs have been investigated, and there are many encouraging results [49]. More human clinical trials are required to confirm the anticancer effects of phytochemical factors, but their anticancer potential should not be underestimated.

## Figures and Tables

**Table 1:** Main anticancer mechanism regulated by natural phytochemicals in oral cancer.

Phytochemicals	Model	Anticancer Mechanism	References
BRBs	cell lines	- inhibition of cell proliferation	[263]
		- inhibition of translation of VEGF	
		- inhibition of nitric oxide synthase activity	
		- induction of apoptosis and terminal differentiation	
	DBP-induced oral cancer model	- enhancing removal of the DBP–DNA adducts	[205,264,265]
		- enhancing the methylation of genes while hypomethylated by DBP: *Fgf3*, *Qrich2*, *Rmdn2*, and *Cbarp*	
		- suppression of the methylation of genes while hypermethylated by DBP: *Vamp3*, *Ppp1r13l*, *Pkm,* and *Zfp316*	
	4NQO-induced oral cancer model	- suppression of the mRNA expression of pro-inflammatory biomarkers (*Cxcl1*, *Mif*, and *Nfe2l2*)	[203,207,208]
		- suppression of the mRNA expression of anti-apoptosis and cell cycle associated markers (*Birc5*, *Aurka*, *Ccna1*, and *Ccna2*)	
		- modulation of glycolysis metabolic pathways	
	DMBA-induced oral cancer model	- inhibition of DNA adducts	[204,266]
		- induction of *RB1* expression	
	clinical trial	- suppression of the mRNA expression of pro-inflammatory biomarkers (*NFKB1* and *PTGS2*) and pro-survival biomarkers (*AURKA*, *BIRC5*, and *EGFR*)	[202,208,209,210]
		- suppression of LOH events (*INK4a/ARF*, *p53*, and *FHIT*)	
		- suppression of genes associated with RNA processing and growth factor recycling	
		- inhibition of apoptosis	
		- suppression of COX-2 expression	
		- suppression of microvessel density	
Green tea, EGCG, EGC, ECG	cell lines	- inhibition of cell proliferation	[141,211]
		- inhibition of migration and invasion	
		- induction of apoptosis and cell cycle arrest	
		- modulate NF-κB and AP-1	
	(1) 4NQO-induced oral cancer model (2) DMBA-induced oral cancer model	- inhibition of oxidative stress and phase I enzymes	[141,267]
		- induction of phase II enzyme activities	
	a mouse xenograft model.	- inhibition of invasion and MMP-9 expression	[214]
		- inhibition of tumor cell growth	
	clinical trial	- inhibition of DNA damage and cell growth	[268]
		- a decrease in cell number in the S-phase of cell cycle	
		- cells accumulated in G1 phase, DNA content became more diploid and less aneuploidy	
		- induction of apoptosis	
Curcumin	cell lines	- inhibition of cell proliferation, invasive ability, and EMT	[222,269]
		- inhibition of the expression of MMP-2 and MMP-9	
		- modulating p53-E-Cadherin and Wnt/β-catenin pathway	
		- induction of apoptosis	
		- up-regulation of C/EBPa and IGFBP-5	
		- induction of miR-9 expression	
	4NQO-induced oral cancer model	- inhibition of cell proliferation	[270]
	(1) DMBA-induced oral cancer model (2) MAOMN-induced oral mucosal tumors	- inhibition of the tumor burden and tumor incidence	[223,224]
	clinical trial	- reduced the lesion size and pain.	[225]
Garlic	cell lines	- inhibition of cell proliferation	[229]
		- induction of the expression of E-cadherin	
		- stabilized the E-cadherin/β-catenin adherent junction complex	
	DMBA-induced oral cancer model	- modulating lipid peroxidation and enhancing the levels of GSH, GPx, and GST	[230,231,232]
		- downregulation of Bcl-2 and upregulation of Bax, Bim, P53, caspases 8, and caspases 3 (the combined administration of tomato and garlic)	
	a mouse xenograft model.	- inhibition of N-methylpurine DNA glycosylase and osteopontin (OPN)	[228]
		- inhibition of the phosphorylation of Akt, mTOR, IκB and Erk1/2	
		- inhibition of the expression of cyclin D1, COX-2, vimentin, and NF-κB p65 (RelA)	
		-induction of the expression of p16Ink4 expression	
Onion	cell lines	- inhibition of cell proliferation, migration, and invasion	[234,236]
		- inhibition of the expression and activity of MMP-2 and MMP-9	
		- inhibition of NF-κB signaling pathways	
		- induction of the cytotoxic effects	
	DMBA-induced oral cancer model	- delay tumor formation	[235]
Resveratrol	cell lines	- inhibition of cell growth and DNA synthesis	[241,242]
		- induction of DNA damage and apoptosis	
	a mouse xenograft model	- inhibition of cell growth and proliferation	[242]
		- induction of DNA damage and apoptosis	
Lycopene	cell lines	- inhibition of cell proliferation	[250,271]
		- enhanced gap-junction communication	
		- induction of Cx43 expression	
	DMBA-induced oral cancer model	- modulated lipid peroxidation	[251]
		- enhanced the activities of the enzymes in the glutathione redox cycle	
Xanthophylls (astaxanthin and canthaxanthin)	4NQO-induced oral cancer model	- inhibition of proliferation	[257]
Bromelain	cell lines	- induction of PARP, cleavage products, and lamin A/C degradation	[261]
		- induction of the sub-G1 population	

Akt: AKT serine/threonine kinase 1; AURKA: aurora kinase A; Birc5: baculoviral IAP repeat containing 5; BRBs: black raspberries; Cbarp: CACN subunit beta associated regulatory protein; C/EBPa: CCAAT enhancer binding protein alpha; Ccna1: cyclin A1; Ccna2: cyclin A2; COX-2: cyclooxygenase-2; Cxcl1: C-X-C motif chemokine ligand 1; DBP: dibenzo-[a,l]-pyrene; Erk1/2: extracellular signal-regulated protein kinases 1 and 2; Fgf3: fibroblast growth factor 3; FHIT: fragile histidine triad diadenosine triphosphatase; GPx: glutathione peroxidase; GSH: glutathione; GST: glutathione s-transferase; IGFBP-5: insulin-like growth factor-binding protein-5; IκB: inhibitor of NF-κB; Mif: macrophage migration inhibitory factor; mTOR: mechanistic target of rapamycin kinase; NF-κB: nuclear factor-κB; Nfe2l2: nuclear factor, erythroid 2 like 2; NFKB1:nuclear factor kappa b subunit 1; 4NQO: 4-nitroquinoline 1-oxide; AP-1: activator protein-1; ECG: (−)-epicatechin-3-gallate; EGC: (−)-epigallocatechin; EGCG: (–)-epigallocatechin-3-gallate; EGFR: epidermal growth factor receptor; EMT: epithelial–mesenchymal transition; MAOMN: methyl-(acetoxymethyl)-nitrosamine; MMP: matrix metalloproteinase; p53: tumor protein P53; PARP: poly (ADP-ribose) polymerase; Pkm: pyruvate kinase muscle isozyme; Ppp1r13l: protein phosphatase 1 regulatory subunit 13 like; PTGS2: prostaglandin-endoperoxide synthase 2; Qrich2: glutamine rich 2; RB1: retinoblastoma gene; Rmdn2: regulator of microtubule dynamics 2; VAMP3: vesicle associated membrane protein 3; VEGF: vascular endothelial growth factor; Zfp316: zinc finger protein 316.

## Abbreviations

4NQO: 4-nitroquinoline 1-oxide; AP-1: activator protein-1; BaP: benzo[a]pyrene; BRBs: black raspberries; CSCs: cancer stem cells; CT: computed tomography; DBP: dibenzo-[a,l]-pyrene; ECG: (−)-epicatechin-3-gallate; EGC: (−)-epigallocatechin; EGCG: (–)-epigallocatechin-3-gallate; EGFR: epidermal growth factor receptor; EMT: epithelial–mesenchymal transition; ERs: estrogen receptors; HBP: hamster buccal pouch; HPV: human papillomavirus; IARC: International Agency for Research on Cancer; LOH: loss of heterozygosity; MAOMN: methyl-(acetoxymethyl)-nitrosamine; miRNAs: microRNAs; MMPs: matrix metallopeptidases; MRI: magnetic resonance imaging; NF-κB: nuclear factor-κB; NNK: 4-(methylnitrosamino)-1-(3-pyridyl)-1-butanone; NNN: N’-nitrosonornicotine; OPL: oral premalignant lesions; OSCC: oral squamous cell carcinoma; PAHs: polycyclic aromatic hydrocarbons; PET: positron emission tomography; SAC: S-allylcysteine; TGF-α: transforming growth factor-α; TSNAs: tobacco-specific nitrosamines; VEGF: vascular endothelial growth factor.
